# Quality Assessment of Apple and Grape Juices from Serbian and German Markets by Planar Chromatography—Chemometrics

**DOI:** 10.3390/molecules27123933

**Published:** 2022-06-19

**Authors:** Đurđa Krstić, Petar Ristivojević, Filip Andrić, Dušanka Milojković-Opsenica, Gertrud E. Morlock

**Affiliations:** 1 University of Belgrade—Faculty of Chemistry, Chair of Analytical Chemistry, Center for Excellence for Molecular Food Sciences, Studentski Trg 12-16, 11158 Belgrade, Serbia; djurdjakrstic@chem.bg.ac.rs (Đ.K.); ristivojevic@chem.bg.ac.rs (P.R.); andric@chem.bg.ac.rs (F.A.); dusankam@chem.bg.ac.rs (D.M.-O.); 2 Justus Liebig University Giessen, Institute of Nutritional Science, Chair of Food Science, and Interdisciplinary Research Center, Heinrich-Buff-Ring 26-32, 35392 Giessen, Germany

**Keywords:** high-performance thin-layer chromatography, HPTLC fingerprint, effect-directed analysis, authenticity, adulteration, falsification

## Abstract

The high consumption of plant-based foods on a global scale has increased the number of adulterations in the food industry. Along with this, analytical approaches to fraud detection need to be further developed. A nontargeted effect-directed profiling by high-performance thin-layer chromatography hyphenated with five effect-directed assays (free radical scavenging assay, *Aliivibrio fischeri* bioassay, and acetylcholinesterase, butyrylcholinesterase, and tyrosinase inhibition assays) and multi-imaging provided additional information on the antioxidative, antimicrobial, and enzyme inhibition activities for 18 apple and 18 grape juices from markets in Serbia and Germany. Bioactive zones of interest were eluted using an elution head-based interface and further characterized by electrospray ionization high-resolution mass spectrometry. The different profiles were evaluated chemometrically, and several compounds, which were characteristic of samples from different markets located in Serbia and Germany, were identified in apple juice (such as chlorogenic acid, phloridzin, epicatechin, and caffeic acid) and grape juice (such as chlorogenic acid, epicatechin, and quercetin). The developed rapid and simple method for the quality assessment of fruit juices coming from different (geographic) markets showed clear quality differences. Thus, it could be used to learn more about quality differences, to detect fraud in fruit juice production, and to verify the authenticity of the origin.

## 1. Introduction

Due to the high nutritional value and bioactive potential of constituents, the fruit juice market has grown in recent years. According to the Global Food Safety Initiative, fruit products sold on the market should be authentic, which means that the declaration on the label should be consistent with the composition and origin of the food ingredients [1]. Commercial fruit juices are obtained by the industrial processing of fruits, and their quality depends on the fruit species, geographical origin, growing conditions, stage of ripeness, and technology used [2]. Fruit juices are often adulterated by misrepresenting the proper species or geographical origin of the fruit or by adding cheaper and inferior ingredients, which leads to a reduction in product quality.

The consumption of apple and grape juices is associated with various health-promoting effects (such as anticancer, antimutagenic, antiproliferative, anti-inflammatory, antimicrobial, and free radical scavenging activities) due to the high content of phenolic compounds [3,4,5]. The most common phenolic constituents in apples are chlorogenic acid, caffeic acid, catechin, epicatechin and their oligomers (proanthocyanidins), quercetin and its glycosides, and dihydrochalcones (such as phloridzin and phloretin xyloglucoside) [6]. On the one hand, phloridzin as a characteristic flavanoid for apples can be used to detect the adulteration of fruit juices [7]. On the other hand, anthocyanins (such as cyanidin, peonidin, delphinidin, petunidin, and malvidin glucosides), resveratrol, flavonols, phenolic acids, and tannins (such as catechins, epicatechins, and proanthocyanidins) can be the main phenolic compounds present in red grapes and juices [8,9,10].

Serbia and Germany are countries with a long tradition of fruit cultivation due to favorable climate conditions. Apple juice is one of the most consumed fruit juices in Serbia and Germany. According to the research on orchards conducted in Serbia and Germany in 2017, apples are in the first place in terms of production and average yield per hectare (https://www.stat.gov.rs; https://apps.fas.usda.gov, accessed on 1 June 2022). Red grape juices have a proven high phenolic content and strong antioxidant activity that make them a preferable option among other widely consumed juices [2].

Due to increasing consumption, the quality assessment of fruit juices has recently become an important issue in the food industry. The quality of fruit juices has a significant influence in terms of consumer protection and food safety. Apple and grape juices have been recognized as functional superfoods in numerous studies. Apple juice had a positive effect on plasmatic antioxidant capacity [11], the prevention of Alzheimer’s disease, and the reduction in the risk of cancer and stroke [12]. It has also been reported that the consumption of grape juice alters oxidative status and may prevent cardiovascular diseases, atherosclerosis, Parkinson’s disease, and cataracts [13,14,15]. Neurocognitive functions can also be improved by consuming grape juice [16]. In addition to biological activity, phenolic compounds also influence the sensory properties of fruit juices, such as color, stability, bitterness, and astringency [9,17]. Evaluation of the unique phenolic profile of fruit juices could be used as a tool for authenticity and the identification of fruit beverages [17]. Investigating the complex phytochemical profile of fruit juices requires the development of new reliable, efficient, and sensitive analytical methods or the improvement of existing ones. The phytochemical profile of geographically different juice samples could be used to build fruit juice databases, which in turn can be used to establish the authenticity and geographical origin of food products by comparing the compositions of unknown samples with reference or control samples.

The most frequently used analytical methods are targeted and focus on the detection and identification of particular compounds or class of compounds as chemical markers for authenticity assessment (such as amino acids, organic acids, saccharides). Target analyses by high-performance liquid chromatography [9] and gas chromatography-quadrupole mass spectrometry [18] are most commonly used to ensure the authenticity of fruit juices, with emphasis on the identification and quantification of specific compounds. High-performance thin-layer chromatography (HPTLC) is becoming more popular in food analysis due to the straightforward hyphenation with planar-effect-directed assays, multi-detection of the same chromatogram, highly targeted substance identification, parallel analyses under the same experimental conditions, low running costs, low solvent consumption [19,20,21], and method greenness compared to HPLC [22]. The obtained HPTLC chromatogram can be statistically analyzed by sophisticated chemometric tools to gain maximum information, such as the identification of chemical markers of geographical origin and similarity/dissimilarity between samples [22,23,24].

The aim of this study was to develop a fast and simple analytical approach for the quality assessment of commercially available apple and grape juices purchased on different markets. The juices were purchased from the local stores in Serbia and Germany and were considered as samples originating from two different market groups. It was of interest to find out if there are differences between the selected markets from Serbia (SMS) *versus* Germany (SMG) with regard to the juice quality. Not only the physicochemical composition, but also the biofunctional composition (bioactivity) of the fruit juices was taken into account. The developed HPTLC method was hyphenated with five different effect-directed assays, having no target analytes in mind. In this effect-directed nontarget analysis, interesting bioactive compounds were observed and further characterized by electrospray ionization–high-resolution mass spectrometry (ESI–HRMS). Sophisticated chemometric techniques were used to learn more about quality differences between two market groups and to identify compounds that are potential markers for the geographic discrimination of apple and grape fruit juices on the markets.

## 2. Results

### 2.1. HPTLC−FLD Profiling of Fruit Juice Extracts

**Figure 1:**
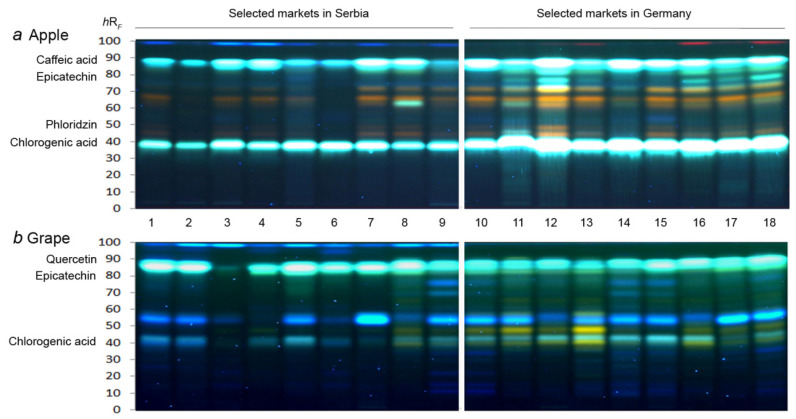
HPTLC−FLD profiles at 366 nm after derivatization with natural product A reagent of extracts of (**a**) apple and (**b**) grape juices from selected markets in Serbia (1−9) and Germany (10−18).

An HPTLC−UV/Vis/FLD method was developed to investigate the profiles of 18 apple and 18 grape juice extracts, whereby 9 samples were collected from markets, each in Serbia and Germany (Table S1). The juice samples were extracted with ethyl acetate–dichloromethane 3:1, concentrated to dryness, and redissolved in methanol. For the first time, a chemical profiling of phenols in apple and grape juices was developed using ethyl acetate–toluene–formic acid–water 16:4:3:2, *V*/*V*/*V*/*V,* as mobile phase. For detection of phenolics, the derivatization with the natural product A reagent (2-aminoethyldiphenylborinate/polyethylene glycol 400) followed. Already this simple physicochemical profiling (providing UV/Vis/FLD chromatograms) showed interesting differences. The chromatogram at fluorescence detection (FLD) 366 nm especially highlighted that the phenolics after derivatization are suited for the differentiation of samples from both markets. A characteristic blue, fluorescent band at *hR*_F_ 98 was present only in the SMS samples in both apple and grape juice extracts (Figure 1). The investigated apple juice extract numbers (nos.) 1−18 (Figure 1a) contained characteristic blue fluorescent bands at *hR*_F_ 37 and 87, as well as orange fluorescent bands at *hR*_F_ 43, 48, 66, and 70. Almost all SMG apple juice samples (nos. 10−18) contained another blue fluorescent band at *hR*_F_ 80, although with different signal intensities, in contrast to the SMS apple juice extracts. The latter showed a lower intensity for the blue fluorescent band at *hR*_F_ 37 in general and at *hR*_F_ 87 in sample nos. 2, 5, 6, and 9. The highest number of well-separated high intense bands at *hR*_F_ 36, 43, 47, 60, 64, 70, and 86 was noticed in SMS sample nos. 7 and 8 and SMG sample nos. 11, 12, and 15.

In the case of the 18 grape juice extract nos. 1−18 (Figure 1b), two blue fluorescent bands at *hR*_F_ 43 and 55 as well as one turquoise fluorescent band at *hR*_F_ 85 were present in almost all analyzed extracts, but with different signal intensities. In addition, an intense orange fluorescent band at *hR*_F_ 48 and further ones were detected in grape juice extract nos. 8−13 and 16−18. The highest number of well-separated high-intensity bands was noticed in sample nos. 8 and 9 from the SMS and 10, 11, and 13 from the SMG.

### 2.2. HPTLC−EDA Profiling of Fruit Juice Extracts

#### 2.2.1. Planar Free Radical Scavenging Assay

**Figure 2:**
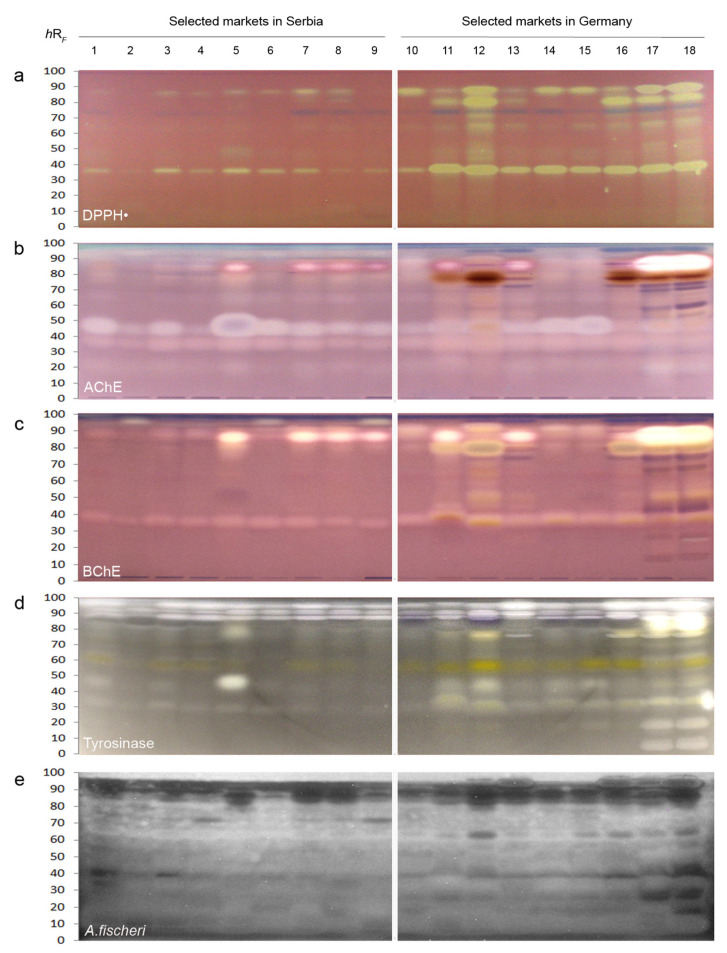
HPTLC profiles of extracts of apple juices from selected markets in Serbia and Germany after the (**a**) DPPH^•^ assay, (**b**) acetylcholinesterase (AChE), (**c**) butyrylcholinesterase (BChE), and (**d**) tyrosinase inhibition assays, all detected under white light illumination, and (**e**) after the *Aliivibrio fischeri* bioassay with the recorded bioluminescence depicted as greyscale image.

**Figure 3:**
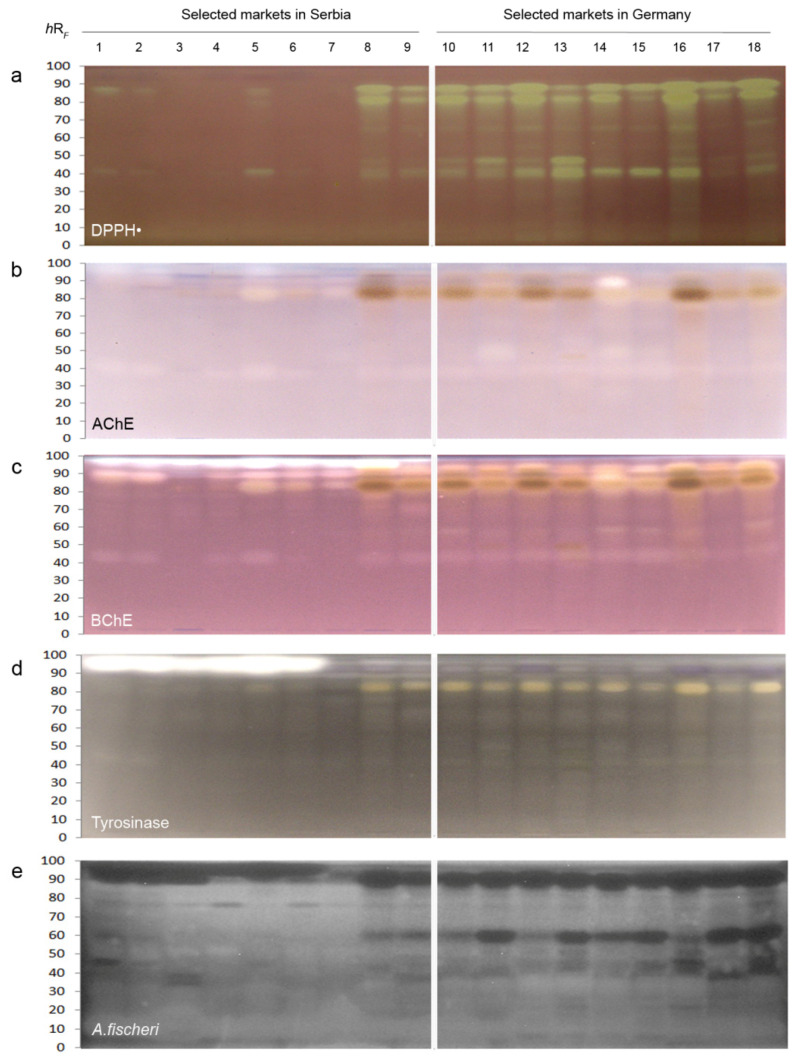
HPTLC profiles of extracts of grape juices from selected markets in Serbia and Germany after the (**a**) DPPH**^•^** assay, (**b**) acetylcholinesterase (AChE), (**c**) butyrylcholinesterase (BChE), and (**d**) tyrosinase inhibition assays, all detected under white light illumination, and (**e**) after the *Aliivibrio fischeri* bioassay with the recorded bioluminescence depicted as greyscale image.

HPTLC chromatograms were dipped in the DPPH**^•^** assay solution to detect compounds with free radical scavenging activity as yellow bands on a purple plate background. Both apple and grape juice extracts possessed DPPH**^•^** scavenging activity (Figure 2a and Figure 3a), whereby nos. 12 and 16 were the most active ones, respectively.

Fruit juice extracts from the SMG (nos. 10−18) showed more active bands than from the SMS (nos. 1−9). The main DPPH**^•^**-active bands of apple juice extracts were observed at *hR*_F_ 37, 48, 64, 80, and 86 (Figure 2a), while for grape juice extracts, these were evident at *hR*_F_ 37, 48, 54, 65, 82, and 90 (Figure 3a).

#### 2.2.2. Planar Enzyme Inhibition Assays

The AChE is the main catalytic enzyme found in synaptic clefts of the central nervous system, erythrocytes, and brain, while the BChE is a nonspecific enzyme, mainly present in the plasma, liver, and muscle tissue [25]. Both are involved in the termination of impulse transmission at cholinergic synapses and related to different neurological disorders, such as Alzheimer disease, ataxia, senile dementia, and myasthenia gravis [26]. Thus, the role of AChE inhibitors is to enhance muscle contraction and strengthen cholinergic neurotransmission. In the HPTLC−AChE/BChE inhibition autograms, AChE (Figure 2b and Figure 3b) and BChE inhibiting compounds (Figure 2c and Figure 3c) were evident as colorless or bright bands against a purple plate background in all analyzed apple and grape juice samples, but their signal intensities were different. Apple samples (Figure 2b) showed a high inhibiting potential with strong AChE inhibiting bands at *hR*_F_ 35, 46 (in no. 5), and 87 (in nos. 17 and 18). In contrast, grape juices had less intensive active bands at *h*R*_F_* 39, 52, 84, and 90 (Figure 3b). The characteristic AChE inhibiting band at *hR*_F_ 39 was noticed in almost all juice extracts.

Characteristic BChE inhibiting bands were observed in all investigated juice extracts. The most active bands among the apple juice extracts were found at *hR*_F_ 35, 80, and 87 (Figure 2c), while the overall highest inhibiting potential was noticed for sample nos. 11, 12, 17, and 18, followed by sample nos. 5, 7–9, 11, and 13. All grape juice extracts contained BChE inhibiting bands at *hR*_F_ 42 and 85, but their intensity varied. The most active band was noticed at *hR*_F_ 96 in juice sample nos. 1–9 from the SMS (Figure 3c).

Tyrosinase is a polyphenol oxidase enzyme, involved in the synthesis of melanin of humans and responsible for the enzymatic browning of fruits and vegetables. Its inhibition is of interest to the food industry to prevent the undesirable browning of foods, and to medicine and cosmetics for the treatment of dermatological problems [27]. In the HPTLC–tyrosinase autograms, tyrosinase inhibitors were evident as colorless or bright bands against a gray background. Among the apple juice extracts (Figure 2d), a similar tyrosinase inhibiting potential was observed at *hR*_F_ 38, 52, 62, 79, and 86. However, nos. 5, 11, 12, 17, and 18 were highlighted due to their overall higher tyrosinase response. In the case of the grape juice extracts (Figure 3d), the already noticed inhibiting band originating from a compound at *hR*_F_ 82 was found in sample nos. 8−18, while samples from the SMS (nos. 1−6) revealed a very strong inhibiting effect for the compound band at *hR*_F_ 95.

#### 2.2.3. Planar Antimicrobial Bioassay

The marine *A. fischeri* bacteria are nonpathogenic and manageable microorganisms for testing the bioactivity of samples [28]. Bioactive compounds with a negative impact on the *A. fischeri* metabolism were detected as dark zones against a bioluminescent background (Figure 2e and Figure 3e, depicted as greyscale image). In all investigated apple juice extracts, compounds at *hR*_F_ 95 and 88 showed the most intense, darkest bands. Apple juice extract no. 18 showed the highest response against *A. fischeri* bacteria, followed by sample nos. 1, 3, 12, 15, 16, and 17. In the analyzed grape juice extracts, sample nos. 8−18 showed a moderate to strong antibacterial activity for bands at *hR*_F_ 90 and 58. The grape juice extracts had stronger antimicrobial activities compared to the apple juice extracts.

### 2.3. Tentative Assignment of Bioactive Compounds in Juice Extracts by HPTLC–ESI–HRMS

As an example for further characterization of bioactive bands in the juice extracts, HPTLC–ESI–HRMS was employed, eluting the bioactive bands via an elution head-based interface into the HRMS. Apple juice sample no. 12 and grape sample no. 14 were selected based on the HPTLC–EDA results. The structural assignment was performed based on the obtained exact masses, isotopes, mass error, and molecular fragments. The strongly blue fluorescent bioactive band at *hR*_F_ 40 (Figure 1a) was tentatively assigned as chlorogenic acid (5-*O*-caffeoylquinic acid), which revealed the deprotonated molecule at *m*/*z* 353.0880 [M−H]^−^ and its characteristic fragment ion at *m*/*z* 191.0562 [M−H−caffeoyl]^−^ (Figure S1a) [29]. Chlorogenic acid was found especially in all apple juice extracts and most grape juice extracts.

The orange fluorescent (after derivatization) bioactive band at *hR*_F_ 50 (Figure 1a) was tentatively assigned to the most prominent apple polyphenol phloridzin (phloretin 2′-*O*-glucoside) [30] based on the HPTLC–ESI–HRMS spectrum (Figure S1b). Its principal biological functions are the regulation of glucose absorption and excretion, inhibition of lipid peroxidation, as well as inhibition of cancer cell growth [31]. Europeans consume an average of 0.7–7.5 mg/day of phloridzin, whose main source are apples and apple juice [32]. The deprotonated molecule at *m*/*z* 435.1299 [M−H]^−^ led to the characteristic phloretin aglycon fragment ion at *m*/*z* 273.0856 (Figure S1b) [33,34].

The blue fluorescent (after derivatization) bioactive band at *hR*_F_ 80 both in the apple and grape juices extracts (Figure 1) showed the deprotonated molecule at *m*/*z* 289.0721 [M−H]^−^ (Figure S1c) and was tentatively assigned as epicatechin. This flavan-3-ol is commonly present in tea plant leaves, but also in fruits, especially grapes and apples. Flavan-3-ols provide a wide range of health beneficial effects by acting as antioxidant, anticarcinogen, cardiopreventive, antimicrobial, anti-viral, and neuro-protective agents [5,35,36].

The blue fluorescent bioactive band at *hR*_F_ 90 (Figure 1a) showed the deprotonated molecule at *m*/*z* 179.0349 [M−H]^−^ (Figure S1d) that corresponds to the caffeic acid and was found in apple juice extract. The caffeic acid fragmentation pathway is based on the loss of the CO_2_ group (–44 Da), resulting in the detected fragment [M–H–CO_2_]. In the analyzed grape juice extract, another deprotonated molecule was evident at *m*/*z* 301.0355 [M−H]^−^ (Figure S1e), which was tentatively assigned to be quercetin.

### 2.4. Multivariate Analysis of HPTLC–FLD and HPTLC–EDA Fingerprints

Principal component analysis (PCA) is the most used pattern recognition technique that allows visualization of the data structure and identification of variables with the highest influence on the classification and differentiation of objects. It was applied on datasets obtained by image analysis of the HPTLC chromatograms to obtain basic insight into the specific grouping patterns and to distinguish apple and grape juice samples from the SMG *versus* SMS. The obtained PCA models are presented in Table S2.

#### 2.4.1. Apple Juices

**Figure 4:**
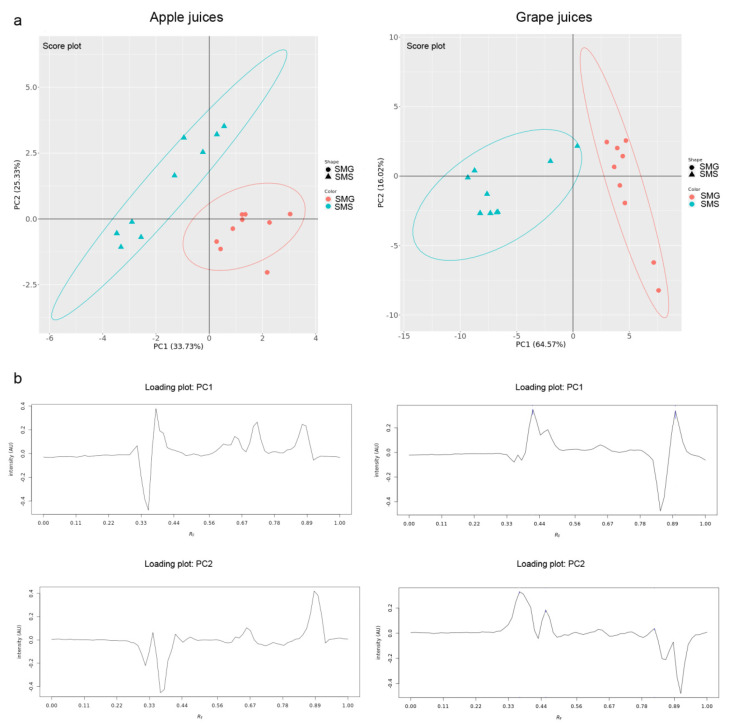
PCA showing the (**a**) PCs score plots and (**b**) PC1 and PC2 loading plots obtained from the HPTLC chromatogram at 366 nm of the extracts of apple and grape juices after derivatization with the natural product A reagent.

For the apple juice extracts, the PCA model applied on the HPTLC−FLD chromatograms at 366 nm after derivatization with the natural product A reagent (highlighting phenolic compounds) displayed a good discrimination between samples collected in Serbia and Germany according to the PC1 axis. Samples from the SMG were positioned at the lower right side of the PCA score plot, while the SMS samples were scattered at the left side of the plot (Figure 4). Within the SMS group two subgroups can be noted along the PC2 direction. The obtained loading plot showed the influence of phenolic compounds with characteristic *hR*_F_ values on the classification of samples according to the geographical market. The compounds at *hR*_F_ 39, 72, and 87 affected PC1 in a positive manner, indicating a higher content of these phenolic compounds in apple juices collected from the SMG, while the compound at *hR*_F_ 36 significantly affected PC1 in a negative manner and is responsible for the differentiation of SMS apple juices. Variables that had the highest positive impact on the differentiation of samples along the PC2 axis were compounds at *hR*_F_ 35, 66, and 88, while compounds at *hR*_F_ 30 and 38 had a negative impact.

In the case of the HPTLC−DPPH**^•^** autograms (Figure S2a), two groups of objects could be noticed (SMG and SMS samples). Variables at *hR*_F_ 48 and 75 (along PC1) as well as at *hR*_F_ 45 and 50 (along PC2) had the highest impact on sample grouping. 

The PCA model obtained for the HPTLC−AChE autograms (Figure S2b) confirmed the differentiation of the samples according to their geographical market. Two samples from the SMS formed a subgroup between clusters belonging to the SMS and SMG apple juices. The loading plots revealed that the compounds at *hR*_F_ 27, 82, and 93 had the most positive impact on the PC1 direction and were responsible for the separation of German apple juices, while the compound at *hR*_F_ 44 mainly affected the differentiation of apple juices from SMS along the PC1 axis. The compounds at *hR*_F_ 25, 53, and 96 had the highest negative impact on separation along PC2, while the compounds at *hR*_F_ 42 and 90 had a strong positive impact. 

In the case of the HPTLC−BChE autograms (Figure S2c), the score plot showed that juice extracts from SMS formed a cluster at the upper side of the plot, while those from SMG were scattered at the lower side of the plot. The highest positive contribution to separation along the PC1 axis had the compound at *h*R*_F_* 85, while the compounds at *hR*_F_ 42, 73, and 95 had the highest negative impact. The differentiation of samples along the PC2 axis was influenced by variables at *hR*_F_ 73, 78, and 96 (in positive manner) and at *hR*_F_ 38 and 92 (in negative manner).

The score plot for the HPTLC−tyrosinase autograms (Figure S2d) revealed a difference in the inhibition potential of SMS and SMG apple juice extracts. SMS samples were grouped at the lower side of the plot, while SMG samples formed the second cluster at the upper side of the plot, along the PC2 axis (except for the two samples that showed a different tyrosinase inhibiting potential from all other samples). Variables at *hR*_F_ 42, 52, and 84 positively affected the separation of SMG samples along the PC2 axis, while compounds at *hR*_F_ 34, 60, and 92 had the highest negative impact on the differentiation of samples from SMS. Additionally, phenolic compounds at *hR*_F_ 45 and 90 significantly affected the PC1 in a positive manner and were responsible for the separation of two German apple juices from all the other analyzed samples. 

The PCA model obtained for the HPTLC−*A. fischeri* bioautograms (Figure S2e) indicated a partial overlapping of SMS and SMG samples, according to the antimicrobial activity. The most influential variables on the separation along the PC1 axis were compounds at *hR*_F_ 84 and 97.

#### 2.4.2. Grape Juices

For the grape juice extracts, the PCA model applied on the HPTLC–FLD chromatogram at 366 nm after derivatization with the natural product A reagent displayed a good discrimination between samples from the SMS *versus* SMG along the PC1 axis (Figure 4). Two samples from SMS formed a subgroup between these separated clusters. Variables at *hR*_F_ 42 and 90 affected PC1 in a positive manner and were responsible for the grouping of the SMG samples at the right side of the plot. The compounds at *hR*_F_ 36 and 85 had the highest negative contribution to the positioning of the objects at the left side of the plot (SMS juices). 

The score plot for the HPTLC−DPPH**^•^** autograms (Figure S3a) suggested the existence of two distinctive clusters along PC1, belonging to grape juice samples from SMG (at the right side of the plot) and SMS (left side of the plot), while two grape juices from SMS overlapped with the samples from SMG because of their similar DPPH**^•^** response. The loading plot showed that the highest positive contribution to the positioning of the objects on the score plot for PC1 had compounds at *hR*_F_ 30, 76, and 80, while the compounds at *hR*_F_ 47, 90, and 96 had negative contributions. Compounds at *hR*_F_ 26 and 67 had the highest positive impact on separation along PC2, while the compounds at *hR*_F_ 33, 78, and 85 had a strong negative impact. 

The PCA model for the HPTLC−AChE autograms (Figure S3b) suggested along the PC2 direction the existence of two distinctive groups belonging to SMG and SMS samples. Two samples from SMG showed a more similar AChE inhibiting potential as well as DPPH**^•^** response to juice samples from SMG. The most influential compound for discriminating juice extracts from SMS was at *hR*_F_ 73, while the compound at *hR*_F_ 90 mostly affected the separation of juices from SMG. 

In the case of the HPTLC−BChE autograms (Figure S3c), samples from SMS were scattered, while juice samples from SMG formed a compact cluster at the right side of the plot, along the PC1 direction. The loading plot revealed that compounds at *hR*_F_ 85 and 95 had the highest positive effect on the PC1 component, indicating a high content in SMG samples. Additionally, compounds at *hR*_F_ 31 and 84 significantly affected the PC2 in a positive manner, while the compounds at *hR*_F_ 43, 90, and 97 had a strong negative impact on separating SMG samples. 

The score plot for the HPTLC−tyrosinase autograms (Figure S3d) suggested the existence of two distinctive clusters, along the PC1 axis. Three samples from SMS showed different inhibition properties from all the other samples. The compound at *hR*_F_ 87 had the most influence on the differentiation of SMG juices along the PC1 axis, while the compound at *hR*_F_ 97 was discriminating the Serbian extracts. The compound at *hR*_F_ 92 had the highest positive impact on separation along the PC2 axis. 

The PCA model for the HPTLC−*A. fischeri* bioautograms (Figure S3e) confirmed a different antimicrobial potential of SMG and SMS grape samples, except for two SMS samples, which showed more similar characteristics to SMG samples. The compounds at *hR*_F_ 32, 50, 60, and 90 were the most important for the differentiation of grape juices along the PC1 axis.

## 3. Discussion

Fingerprinting methods highlight a number of unique chromatographic signals to enable sample recognition. The visual examination of the chromatograms revealed differences in the profiles among the apple juice extracts originating from SMS and SMG. All analyzed extracts were characterized via a vast number of blue and orange fluorescent bands at *hR*_F_ 36, 44, 47, 65, 77, and 86. The recording of HPTLC−ESI−HRMS spectra allowed the further characterization of interesting zones. In the case of the apple juices, compounds at *hR*_F_ 40, 50, 80, and 90 were assigned as chlorogenic acid (5-caffeoylquinic acid), phloridzin, epicatechin, and caffeic acid, respectively. This is in accordance with published data for German juice made of dessert and cider apples, where chlorogenic acid, quercetin glycosides, procyanidins, and dihydrochalcones, such as phloridzin (phloretin-*O*-glucoside) and phloretin-*O*-xyloglucoside, were found as dominant constituents [6]. In the case of the grape juices, blue fluorescent bands at *hR*_F_ 43, 55, 85, and 90, as well as an orange fluorescent band at *hR*_F_ 48, were present in all analyzed extracts. The bands at *hR*_F_ 43, 85, and 90 were identified as chlorogenic acid, epicatechin, and quercetin, respectively. The characterization of grape juices produced in Brazil showed that anthocyanins and tannins were predominant phenolic compounds [9].

The effect-directed analysis of the investigated fruit juices showed a moderate to strong antioxidant potential as well as AChE, BChE, and tyrosinase inhibitory activities. In the literature [37], similar results were obtained for apple juice from a Chinese market, which confirmed the strong antioxidant activity of 5-*O*-caffeoylquinic acid, quercetin, and phloretin that significantly increased after juice fermentation with *Lactobacillus plantarum*. The analysis of the antioxidant and antimicrobial melatonin in 18 apple cultivars and juices [38] indicated that the highest melatonin level was detected in the peel, while the melatonin content in the juice was comparable to that of its flesh. Examination of the antioxidant effects of grape skin anthocyanins using various in vitro and in vivo methods confirmed a strong antioxidant and AChE inhibition potential [39]. Antimicrobials against Gram-negative *A. fischeri* were detected in all analyzed juices, but grape juice extracts showed stronger antimicrobial activities compared to apple juices. This is contrary to results obtained by the evaluation of the antibacterial activity of apple, pomegranate, and grape juices on clinical endodontic bacterial strains, where the highest antibacterial activity was observed in apple fruit juice [40]. However, this contradiction may be explained by varying antimicrobial pesticide residues. Evaluation of the antimicrobial effect of white grape juice extract (Trapani, Italy) against a range of Gram-positive and Gram-negative bacteria confirmed that *Staphylococcus aureus* was the most sensitive strain among the tested Gram-positive bacteria, while *Escherichia coli* was the only susceptible strain in the case of Gram-negative bacteria [41].

The proposed untargeted effect-directed profiling of phenolic compounds in fruit juice extracts could be considered as a promising analytical tool in the quality control of fruit juices, but it must be taken into account that fruit variety, ripening stage, environmental conditions, and processing technology also affect the phenolic content of fruit juices [42]. The PCA score plots reveal that the best separation between apple juices of SMG and SMS was achieved by using the HPTLC−FLD chromatogram at 366 nm after derivatization, which was the simplest protocol among the tested detection options. The effect-directed profiles (DPPH**^•^**, AChE, BChE, and tyrosinase inhibition) lead to a comparatively lower degree of separation between the samples of different geographic markets, whereby the BChE inhibition assay was the worst. However, it was still possible to separate the samples sufficiently. A similar degree of separation of apples from five geographic areas (Alpine, Dinaric, Mediterranina, Panonian, and Submetiranian) was achieved using linear discriminant analysis as the classification method and the content of primary metabolites, as well as polyphenols as input [43]. Our excellent separation of samples from two market groups (SMG and SMS) obtained by physicochemical HPTLC profiling can also be compared with the results obtained for five apple cultivars collected from five regions in China (Liquan, Xunyi, Yongshou, Sanyuan, and Luochua) using linear discriminant analysis as the classification method and the content of polyphenols as the input [44]. All this might suggest that the chemical fingerprint related to the polyphenolic content plays a significant role in the discrimination of apple juices according to geographic markets.

In the case of the grape juice samples, the PCA score plots revealed that the HPTLC fingerprints and HPTLC−DPPH**^•^** profiles provided enough information to completely distinguish samples of German and Serbian markets. The separation of different geographic samples based on the HPTLC−tyrosinase inhibition profiles was satisfactory. However, HPTLC−AChE and BChE inhibition profiles did not provide a sufficient separation. The information obtained from the physicochemical composition had a clear advantage over the effect-directed data, regarding the discrimination of apple and grape juice extracts from two selected market groups. Effect-directed data reveal the presence of important bioactive compounds, which may vary more strongly, but provide strong arguments for the valorization of food. Hence, further in-depth effect-directed studies are needed especially for plant-based food.

## 4. Materials and Methods

### 4.1. Reagents and Chemicals

Polyethylene glycol 400, 2-aminoethyldiphenylborinate (97%), Fast Blue Salt B, 2,2-diphenyl-1-picrylhydrazyl radical (DPPH, 97%), butyrylcholinesterase (BChE, from equine serum, ≥140 U/mg) and acetylcholinesterase (AChE, from *Electrophorus electricus* Linnæus, ≥245 U/mg, 10 kU/vial), tyrosinase (from mushroom, ≥1000 U/mg, 25 kU/vial), and solvents/reagents of analytical grade were purchased from Fluka Sigma-Aldrich, Schnelldorf, Germany. Luminescent marine *A. fischeri* bacteria (NRRL-B11177, DSM no. 5171) were obtained from the German Collection of Microorganisms and Cell Cultures. (2S)-2-Amino-3-(3,4-dihydroxyphenyl) propionic acid (levodopa) was obtained from Santa Cruz Biotechnology, Dallas, TX, United States. Ethanol, and methanol and its MS-grade were provided from Fisher Scientific, Schwerte, Germany. Formic acid (96%), toluene, dichloromethane, ethyl acetate, and HPTLC plates silica gel 60 F_254_ were obtained by Merck, Darmstadt, Germany. Bidistilled water was prepared with a Destamat Bi 18E, Heraeus, Hanau, Germany.

### 4.2. Sample Preparation

Fruit juice samples (18 apple and 18 grape juices) were collected from local markets in Belgrade, Serbia (Table S1, nos. 1−9) and Giessen, Germany (nos. 10−18). Therefore, the total number of analyzed samples was 36 (9 samples × 2 market groups × 2 juice types). Each of the 9 samples was considered as belonging to the same population. The fruit juices (100 mL each) were concentrated to 20 mL (basic rotary evaporator RV 05, IKA-Werke, Staufen, Germany) under reduced pressure at 35 °C. Extraction was performed with 20 mL of ethyl acetate–dichloromethane 3:1, *V*/*V*, under agitation for 3 min (Basic Vortex Mixer, Thermo Fisher Scientific, Dreieich, Germany) and repeated once. Both pooled organic phases were evaporated to dryness and each residue was dissolved in 2 mL of methanol prior to storage at −20 °C. In the final experiments, no replicate measurements were performed.

### 4.3. HPTLC–UV/Vis/FLD and HPTLC–EDA–UV/Vis/FLD Analysis

On the HPTLC plate silica gel 60 F_254_ (20 cm × 10 cm, Art. 105641, Merck), aliquots of each fruit juice extract (3 μL for FLD 366 nm, 1 µL for DPPH**^•^** assay, 5 µL for *A. fischeri* bioassay and AChE inhibition assay, and 10 µL for BChE and tyrosinase inhibition assays) were applied as an 8 mm band (Automatic TLC sampler 4). No replicates were performed in the final experiments. HPTLC instruments were from CAMAG, Muttenz, Switzerland and controlled by winCATS software. Development was performed with ethyl acetate–toluene−formic acid−water 16:4:3:2, *V*/*V*/*V*/*V* [45], up to a migration distance of 70 mm in a 10 min saturated twin trough chamber (with filter paper) followed by drying under a cold flow of air (hair dryer) and heating at 100 °C for 3 min (TLC Plate Heater III). Images of the chromatograms were captured at UV/Vis/FLD (DigiStore 2). Respective positive controls were applied as described [36].

For derivatization with the natural product A reagent detecting phenolics as fluorescent bands, HPTLC chromatograms were immediately (still hot) immersed (immersion time 2 s, immersion speed 3.5 cm/s, Chromatogram Immersion Device III, if not stated otherwise) in a 0.5% solution of 2-aminoethyldiphenylborinate in ethyl acetate and then a 5% solution of polyethylene glycol 400 in dichloromethane. The images captured at FLD 366 nm were saved as TIF files.

For the DPPH**^•^** assay detecting radical scavengers as a yellow band against a violet background, HPTLC chromatograms were dipped into a 0.05% methanolic DPPH**^•^** solution [46], dried (60 °C, 1 min), and recorded under white light illumination in the reflectance mode. The images were captured again on the next day, as the signal intensity increased over time.

For the *A. fischeri* bioassay detecting antimicrobials as dark zones against a light grey background (bioluminescence depicted as greyscale image, BioLuminizer), HPTLC chromatograms were immersed into the luminescent Gram-negative *A. fischeri* bacteria suspension, prepared according to [28] and ready for use when a brilliant green-blue bioluminescent light has been emitted when the flask is shaken in the dark. Images were instantly captured with an exposure time of 30 s over a period of 20 min, which allowed time-dependent changes to be monitored.

For the AChE and BChE inhibition assays detecting enzyme inhibitors as colorless or bright bands against a purple background [47], the HPTLC chromatograms were prewetted with 1 mL of Tris-HCl buffer solution (pH 7.8, 0.05 M), immersed in the buffered enzyme solutions (AChE 666 units or BChE 334 units, plus 100 mg of BSA and 100 mL of 0.05 M TRIS buffer, pH 7.8), placed in a moistened plastic box covered with wet filter papers, and incubated at 37 °C for 30 min. Then, plates were immersed into the 1:1 solution of substrate (2.5 mg/mL of ethanolic α-naphthyl acetate solution) and chromogenic reagent (2.5 mg/mL of aqueous Fast Blue Salt B solution), dried, and recorded under white light illumination in the reflectance mode.

For the tyrosinase inhibition assay detecting enzyme inhibitors as colorless or bright bands against a purple background [48], the neutralized HPTLC chromatograms were first sprayed (Derivatizer) with 1 mL of substrate solution (45 mg of levodopa, 25 mg of CHAPS, and 75 mg of PEG 8000 were dissolved in 10 mL of phosphate buffer, consisting of 1.4 mg/mL of dipotassium hydrogen phosphate trihydrate and 1.68 mg/mL of disodium hydrogen phosphate, pH 6.8), then after drying, sprayed with 1 mL of enzyme solution (400 U/mL in phosphate buffer) and incubated in a humid box for 20 min. Images were recorded under white light illumination in reflectance mode.

### 4.4. Characterization of Bioactive Compounds by HPTLC–ESI–HRMS

The bioactive compounds were marked with a soft pencil on the HPTLC plate illuminated at 366 nm and transferred with an elution head-based interface (oval elution head of 4 mm × 2 mm, TLC-MS Interface, CAMAG) with 100% methanol at a flow rate of 0.1 mL/min to the heated ESI source. Full-scan mass spectra (*m/z* 50–750) were recorded at a resolution of 280,000 (FWHM at *m*/*z* 200), AGC target of 1e6, and maximum inject time of 200 ms with lock masses of 301.14103 (dibutyl phthalate, [M+H]^+^) and 413.26623 (diisoctyl phthalate, [M+H]^+^) in the positive and 112.98563 (formic acid, [2M+Na–2H]^–^) in the negative ionization mode via the Q Exactive Plus Hybrid Quadrupole-Orbitrap Mass Spectrometer (Thermo Fischer Scientific, Germany) with a spray voltage of ±3.5 kV, capillary temperature of 270 °C, sheath gas of 20 (arbitrary units), aux gas of 10 (arbitrary units), probe heater temperature of 200 °C, and S-lens RF level of 50 (arbitrary units). A representative plate background at a migration position comparable to the analyte zone was subtracted from the analyte spectrum. Instrument control and data processing were performed using Xcalibur 4.2.47 SP software with Foundation 3.1.261.0 SP6 and SII for Xcalibur 1.5.0.10747 (Thermo Fisher Scientific, Waltham, MA, USA).

### 4.5. Data Acquisition and Statistical Analysis

The open-source software rTLC v1.0 (http://shinyapps.ernaehrung.uni-giessen.de/rtlc/ accessed on 1 June 2022) was used for image processing and multivariate analysis of HPTLC chromatograms. The median filter function with a two-pixel width filter was used for denoising of the images. Normalization of the images was performed by Standard Normal Variate (SNV) transformation, which removes scatter effects by centering each individual variable. The correlation-optimized warping (COW) algorithm was applied in order to remove the negative impact of the band shifts caused by fluctuations in experimental conditions, analyst errors, and instrumental variations. All data were mean-centered to transform variables in the same unit, prior to multivariate analysis.

## 5. Conclusions

The combination of nontarget chemical/biological/biochemical profiling with advanced chemometrics has proven to be an effective tool to learn more about fruit juice quality differences. It is also useful in detecting fraud in fruit juice production (due to the multi-imaging/detection feature and parallel analysis) and in authenticating the geographical origin of apple and grape juices. The bioactivity assessment revealed several characteristic bioactive compounds of apple and grape juices, of which some were tentatively assigned. The examined samples showed moderate antioxidant activity, strong AChE, BChE, and tyrosinase inhibiting potential and good response against *A. fischeri* bacteria. Multivariate data analysis applied on physicochemical and effect-directed profiles clearly showed that there are fruit juice quality differences between countries. Information on the bioactivity of fruit juices obtained through effect-directed profiling expands and improves knowledge about the juice product quality. Hence, this fast and cost-effective analytical approach could find application in the quality assessment of other fruit juices and further fruit products, such as jam and marmalade.

## Data Availability

The data presented in this study are available on request from the authors.

## Supplementary Materials

The following supporting information can be downloaded on https://www.mdpi.com/article/10.3390/molecules27123933/s1, Table S1: List of the analyzed apple and grape juices; Table S2: Statistical performance of the PCA models; Figure S1: HPTLC–ESI–HRMS spectra of the deprotonated molecules of (a) 5-*O*-caffeoylquinic acid at *m/z* 353.0880 [M−H]^−^ and (b) phloridzin at *m/z* 435.1299 [M−H]^−^; Figure S2: PCA score and loading plots of apple juice extracts performed on profile data obtained from the (a) DPPH• assay, (b) AChE, (c) BChE, and (d) tyrosinase inhibition assay as well as (e) *A. fischeri* bioassay autograms; Figure S3: PCA score and loading plots of grape juice extracts performed on profile data obtained from the (a) DPPH• assay, (b) AChE, (c) BChE, and (d) tyrosinase inhibition assay as well as (e) *A. fischeri* bioassay autograms.

## Sample Availability

Food samples are not available from the authors.

## References

[1] Dasenaki M.E., Thomaidis N.S. (2019). Quality and authenticity control of fruit juices-a review. Molecules.

[2] Moreno-Montoro M., Olalla-Herrera M., Gimenez-Martinez R., Navarro-Alarcon M., Rufián-Henares J.A. (2015). Phenolic compounds and antioxidant activity of Spanish commercial grape juices. J. Food Compost. Anal..

[3] Mitić M.N., Obradović M.V., Kostić D.A., Nasković D.Č., Micić R.J. (2011). Phenolics content and antioxidant capacity of commercial red fruit juices. Hem. Ind..

[4] Xia E.Q., Deng G.F., Guo Y.J., Li H.B. (2010). Biological activities of polyphenols from grapes. Int. J. Mol. Sci..

[5] Gerhauser C. (2008). Cancer chemopreventive potential of apples, apple juice, and apple components. Planta Med..

[6] Kahle K., Kraus M., Richling E. (2005). Polyphenol profiles of apple juices. Mol. Nutr. Food Res..

[7] Spinelli F.R., Dutra S.V., Carnieli G., Leonardelli S., Drehmer A.P., Vanderlinde R. (2016). Detection of addition of apple juice in purple grape juice. Food Control.

[8] Cosme F., Pinto T., Vilela A. (2018). Phenolic compounds and antioxidant activity in grape juices: A chemical and sensory view. Beverages.

[9] Natividade M.M.P., Corrêa L.C., de Souza S.V.C., Pereira G.E., de Oliveira Lima L.C. (2013). Simultaneous analysis of 25 phenolic compounds in grape juice for HPLC: Method validation and characterization of São Francisco Valley samples. Microchem. J..

[10] Castillo-Muñoz S., Gómez-Alonso E., García-Romero M.V., Gómez A.H., Velders I., Hermosín-Gutiérrez N. (2009). Flavonol 3-*O*-glycosides series of *Vitis vinifera* Cv. Petit Verdot red wine grapes. J. Agric. Food Chem..

[11] Bitsch R., Netzel M., Carlé E., Strassss G., Kesenheimer B., Herbsbst M., Bitsch I. (2000). Bioavailability of antioxidative compounds from Brettacher apple juice in humans. Innov. Food Sci. Emerg. Tech..

[12] Maragò E., Iacopini P., Camangi F., Scattino C., Ranieri A., Stefani A., Sebastiani L. (2015). Phenolic profile and antioxidant activity in apple juice and pomace: Effects of different storage conditions. Fruits.

[13] Singletary K.W., Stansbury M.J., Giusti M., Van Breemen R.B., Walling M., Rimando A. (2003). Inhibition of rat mammary tumorigenesis by Concord grape juice constituents. J. Agric. Food Chem..

[14] Park Y.K., Lee S.H., Park E., Kim J.S., Kang M.H. (2009). Changes in antioxidant status, blood pressure, and lymphocyte DNA damage from grape juice supplementation. Ann. N. Y. Acad. Sci..

[15] Dani C., Oliboni L.S., Vanderlinde R., Para D., Dias J.F., Yoneama M.L., Bonatto D., Salvador M., Henriques J.A.P. (2009). Antioxidant activity and phenolic and mineral content of rose grape juice. J. Med. Food.

[16] Krikorian R., Boespflug E.L., Fleck D.E., Stein A.L., Wightman J.D. (2012). Concord grape juice supplementation and neurocognitive function in human aging. J. Agric. Food Chem..

[17] Recamales Á.F., Sayago A., González-Miret M.L., Hernanz D. (2006). The effect of time and storage conditions on the phenolic composition and colour of white wine. Food Res. Int..

[18] Perestrelo R., Silva C., Silva P., Medina S., Câmara J.S. (2019). Differentiation of fresh and processed fruit juices using volatile composition. Molecules.

[19] Morlock G.E. (2021). High-performance thin-layer chromatography combined with effect-directed assays and high-resolution mass spectrometry as an emerging hyphenated technology: A tutorial review. Anal. Chim. Acta.

[20] Kirchert S., Kaiser R.E., Morlock G.E. (2019). In-process quality control of wine by planar chromatography versus micro planar chromatography. J. Chromatogr. A.

[21] Krüger S., Urmann O., Morlock G.E. (2013). Development of a planar chromatographic method for quantitation of anthocyanes in pomace, feed, juice and wine. J. Chromatogr. A.

[22] Ristivojević P., Andrić F., Vasić V., Milojković Opsenica D., Morlock G.E. (2022). Fast detection of apricot product frauds by added pumpkin via planar chromatography and chemometrics: Greenness assessment by analytical Eco-Scale. Food Chem..

[23] Ristivojević P.M., Morlock G.E. (2018). Effect-directed classification of biological, biochemical and chemical profiles of 50 German beers. Food Chem..

[24] Fichou D., Ristivojević P., Morlock G.E. (2016). Proof-of-principle of rTLC, an open-source software developed for image evaluation and multivariate analysis of planar chromatograms. Anal. Chem..

[25] Mushtaq G., Greig N.H., Khan J.A., Kamal M.A. (2014). Status of acetylcholinesterase and butyrylcholinesterase in Alzheimer’s disease and type 2 diabetes mellitus. CNS Neurol. Disord. Drug Targets.

[26] Colovic M.B., Krstic D.Z., Lazarevic-Pasti T.D., Bondzic A.M., Vasic V.M. (2013). Acetylcholinesterase inhibitors: Pharmacology and toxicology. Curr. Neuropharmacol..

[27] Kubglomsong S., Theerakulkait C., Reed R.L., Yang L., Maier C.S., Stevens J.F. (2018). Isolation and identification of tyrosinase-inhibitory and copper-chelating peptides from hydrolyzed rice-bran-derived albumin. J. Agric. Food Chem..

[28] (2009). Water Quality—Determination of the Inhibitory Effect of Water Samples on the Light Emission of Vibrio fischeri (Luminescent Bacteria Test)—Part 1: Method Using Freshly Prepared Bacteria.

[29] Marks S.C., Mullen W., Crozier A. (2007). Flavonoid and chlorogenic acid profiles of English cider apples. J. Sci. Food Agric..

[30] Londzin P., Siudak S., Cegieła U., Pytlik M., Janas A., Waligóra A., Folwarczna J. (2018). Phloridzin, an apple polyphenol, exerted unfavorable effects on bone and muscle in an experimental model of type 2 diabetes in rats. Nutrients.

[31] Baldisserotto A., Malisardi G., Scalambra E., Andreotti E., Romagnoli C., Vicentini C.B., Manfredini S., Vertuani S. (2012). Synthesis, antioxidant and antimicrobial activity of a new phloridzin derivative for dermo-cosmetic applications. Molecules.

[32] Niederberger K.E., Tennant D.R., Bellion P. (2020). Dietary intake of phloridzin from natural occurrence in foods. Brit. J. Nutr..

[33] Hvattum E. (2002). Determination of phenolic compounds in rose hip (*Rosa canina*) using liquid chromatography coupled to electrospray ionisation tandem mass spectrometry and diode-array detection. Rapid Commun. Mass Spectrom..

[34] Sánchez-Rabaneda F., Jauregui O., Lamuela-Raventós R.M., Viladomat F., Bastida J., Codina C. (2004). Qualitative analysis of phenolic compounds in apple pomace using liquid chromatography coupled to mass spectrometry in tandem mode. Rapid Commun. Mass Spectrom..

[35] Aron P.M., Kennedy J.A. (2008). Flavan-3-ols: Nature, occurrence and biological activity. Mol. Nutr. Food Res..

[36] Morlock G.E., Heil J., Inarejos-García A.M., Maeder J. (2021). Effect-directed profiling of powdered tea extracts for catechins, theaflavins, flavonols and caffeine. Antioxidants.

[37] Li Z., Teng J., Lyu Y., Hu X., Zhao Y., Wang M. (2018). Enhanced antioxidant activity for apple juice fermented with Lactobacillus plantarum ATCC14917. Molecules.

[38] Zhang H., Liu X., Chen T., Ji Y., Shi K., Wang L., Zheng X., Kong J. (2018). Melatonin in apples and juice: Inhibition of browning and microorganism growth in apple juice. Molecules.

[39] Pervin M., Hasnat M., Lee Y.M., Kim D.H., Jo J.E., Lim B.O. (2014). Antioxidant activity and acetylcholinesterase inhibition of grape skin anthocyanin (GSA). Molecules.

[40] Behera S., Khetrapal P., Punia S.K., Agrawal D., Khandelwal M., Lohar J. (2017). Evaluation of antibacterial activity of three selected fruit juices on clinical endodontic bacterial strains. J. Pharm. Bioall. Sci..

[41] Filocamo A., Bisignano C., Mandalari G., Navarra M. (2015). In vitro antimicrobial activity and effect on biofilm production of a white grape juice (Vitis vinifera) extract. Evid. Based Complement. Altern. Med..

[42] Guo J., Yue T., Yuan Y. (2012). Feature selection and recognition from nonspecific volatile profiles for discrimination of apple juices according to variety and geographical origin. J. Food Sci..

[43] Bat K.B., Vodopivec B.M., Eler K., Ogrinc N., Mulič I., Masuero D., Vrhovšek U. (2018). Primary and secondary metabolites as a tool for differentiation of apple juice according to cultivar and geographical origin. LWT.

[44] Guo J., Yue T., Yuan Y., Wang Y. (2013). Chemometric classification of apple juices according to variety and geographical origin based on polyphenolic profiles. J. Agric. Food Chem..

[45] Jamshidi-Aidji M., Morlock G.E. (2015). Bioprofiling of unknown antibiotics in herbal extracts: Development of a streamlined direct bioautography using Bacillus subtilis linked to mass spectrometry. J. Chromatogr. A.

[46] Pozharitskaya O.N., Ivanova S.A., Shikov A.N., Makarov V.G. (2008). Separation and free radical-scavenging activity of major curcuminoids of *Curcuma longa* using HPTLC-DPPH method. Phytochem. Anal..

[47] Hage S., Morlock G.E. (2017). Bioprofiling of *Salicaceae* bud extracts through high-performance thin-layer chromatography hyphenated to biochemical, microbiological and chemical detections. J. Chromatogr. A.

[48] Morlock G.E., Heil J. (2020). HI-HPTLC-UV/Vis/FLD-HESI-HRMS and bioprofiling of steviol glycosides, steviol, and isosteviol in Stevia leaves and foods. Anal. Bioanal. Chem..

