# Supplementary material for: Quality Assessment of Apple and Grape Juices from Serbian and German Markets by Planar Chromatography—Chemometrics
Source: Molecules. 2022 Jun 19;27(12):3933. doi: 10.3390/molecules27123933 (PMC9230071; doi:10.3390/molecules27123933)
Supplement: Supplementary file 1 [file molecules-27-03933-s001.zip › molecules-1737753-supplementary.pdf]

Supplementary material

Quality assessment of apple and grape juices from Serbian and German markets by planar chromatography–chemometrics

Đurđa Krstić [1], Petar Ristivojević [1], Filip Andrić [1], Dušanka Milojković-Opsenica [1] and Gertrud E. Morlock [2,*]

[1]University of Belgrade – Faculty of Chemistry, Chair of Analytical Chemistry and Center of Excellence for Molecular Food Sciences, Studentski trg 12-16, 11158, Belgrade, Serbia; djurdjakrstic@chem.bg.ac.rs; ristivojevic@chem.bg.ac.rs; andric@chem.bg.ac.rs; dusankam@chem.bg.ac.rs

[2]Justus Liebig University Giessen, Institute of Nutritional Science, Chair of Food Science, and Interdisciplinary Research Center, Heinrich-Buff-Ring 26-32, 35392 Giessen, Germany; gertrud.morlock@uni-giessen.de

*Gertrud Morlock: gertrud.morlock@uni-giessen.de

Content

Table S1. List of the analyzed apple and grape juices.

Table S2. Statistical performance of the PCA models.

Figure S1. HPTLC–ESI–HRMS spectra of the deprotonated molecules of (a) 5-*O*-caffeoylquinic acid at *m/z* 353.0880 $[M-H]^-$ and (b) phloridzin at *m/z* 435.1299 $[M-H]^-$.

Figure S2. PCA score and loading plots of apple juice extracts performed on profile data obtained from the (a) DPPH• assay, (b) AChE, (c) BChE, and (d) tyrosinase inhibition assay as well as (e) *Aliivibrio fischeri* bioassay autograms.

Figure S3. PCA score and loading plots of grape juice extracts performed on profile data obtained from the (a) DPPH• assay, (b) AChE, (c) BChE, and (d) tyrosinase inhibition assay as well as (e) *Aliivibrio fischeri* bioassay autograms.

Table S1. List of the analyzed apple and grape juices.

Apple	Origin of fruit sample
1	
2	
3	
4	Belgrade, Serbia (fruit juices were purchased from local fruit juice shops as randomly chosen commercial products)
5	
6	
7	
8	
9	
10	Germany (Solevita)
11	Germany (*Müller*)
12	Germany (Alnatura)
13	Germany (Lindavia)
14	Germany (Jeden Tag)
15	Germany (REWE)
16	Germany (*dm* Bio)
17	Germany (Amecke)
18	Germany (Tegut)
**Grape**	**Origin of fruit sample**
1	
2	
3	
4	Serbia, Valjevo (fruit juices were purchased from a local fresh fruit juice markets)
5	
6	
7	
8	Belgrade, Serbia (fruit juices were purchased from a local fruit juice shop as randomly chosen commercial products)
9	
10	Germany (Solevita)
11	Germany (*dm* Bio)
12	Germany (Hardthof)
13	Germany (Alnatura)
14	Germany (Tegut)
15	Germany (Lindavia)
16	Germany (Jeden Tag)
17	Germany (REWE Bio)
18	Germany (REWE)

Table S2. Statistical performance of the PCA models.

	PCA model	PCs	% Variance captured by PC1	% Variance captured by PC2	% Variance captured by PC3	% Variance captured by PC4
**Apple**	Fingerprint	4	33.73	25.33	13.58	11.80
	DPPH• assay	5	42.41	15.96	13.06	9.03
	Aliivibrio fischeri bioassay	4	33.57	24.03	12.91	7.43
	AChE inhibition assay	4	49.83	17.68	12.94	6.04
	BChE inhibition assay	5	45.99	21.25	11.88	6.27
	Tyrosinase inhibition assay	4	46.99	27.24	10.74	5.73
**Grape**	Fingerprint	4	64.57	16.02	4.72	3.80
	DPPH• assay	4	59.79	23.07	6.79	3.41
	Aliivibrio fischeri bioassay	4	61.97	14.74	8.39	5.49
	AChE inhibition assay	4	61.09	20.56	5.15	4.14
	BChE inhibition assay	4	36.15	20.77	15.53	10.44
	Tyrosinase inhibition assay	4	59.85	25.97	6.94	2.72

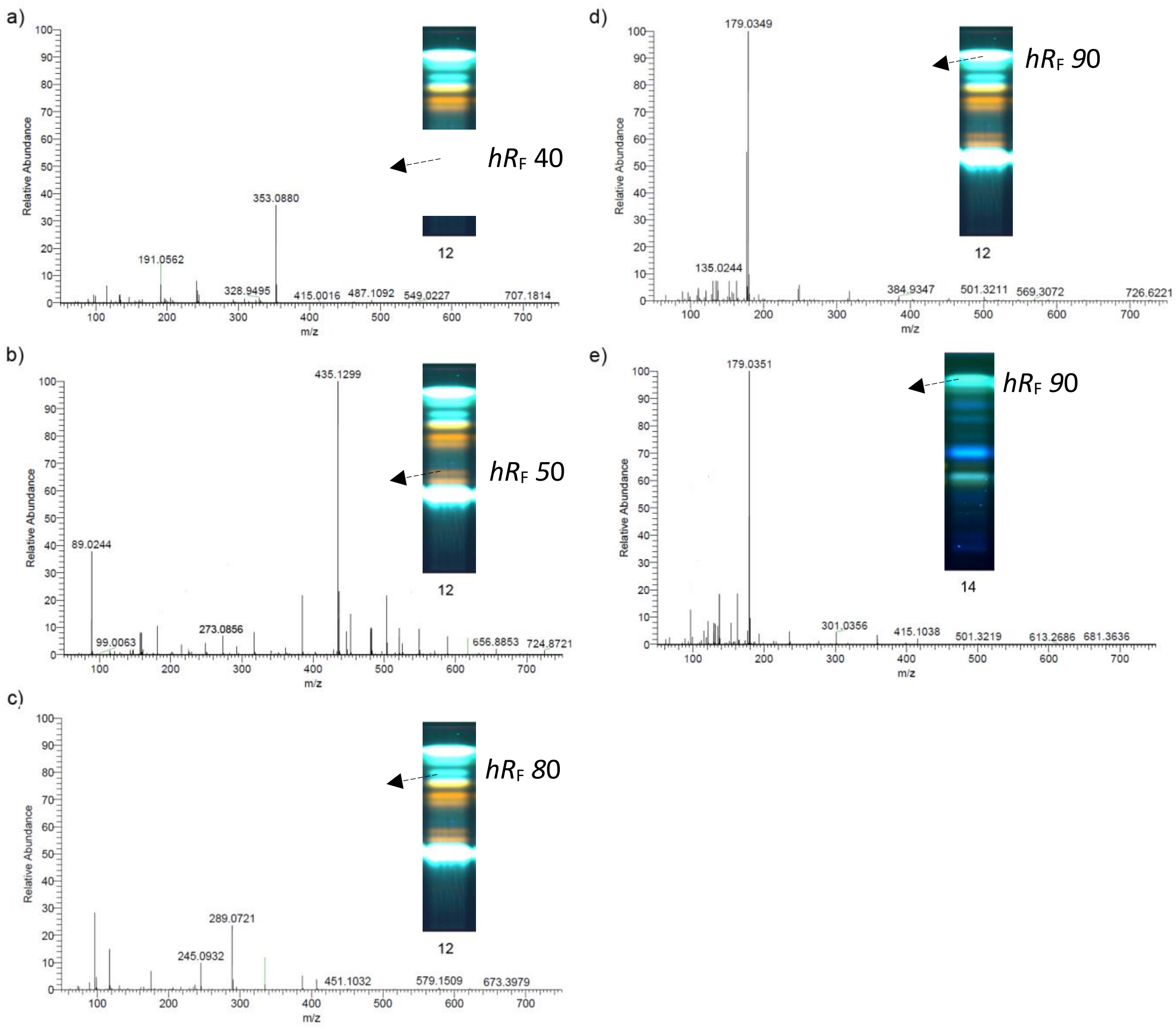

Figure S1. HPTLC–ESI–HRMS spectra of the deprotonated molecules of (**a**) 5-*O*-caffeoylquinic acid at *m/z* 353.0880 $[M-H]^-$, (**b**) phloridzin at *m/z* 435.1299 $[M-H]^-$, (**c**) epicatechin at *m/z* 289.0721 $[M-H]^-$, (**d**) caffeic acid at 179.0349 $[M-H]^-$ in the apple juice sample no. 12 and (**e**) quercetin at 301.0355 $[M-H]^-$ in the grape juice sample no. 14.

Scores	Loadings

a

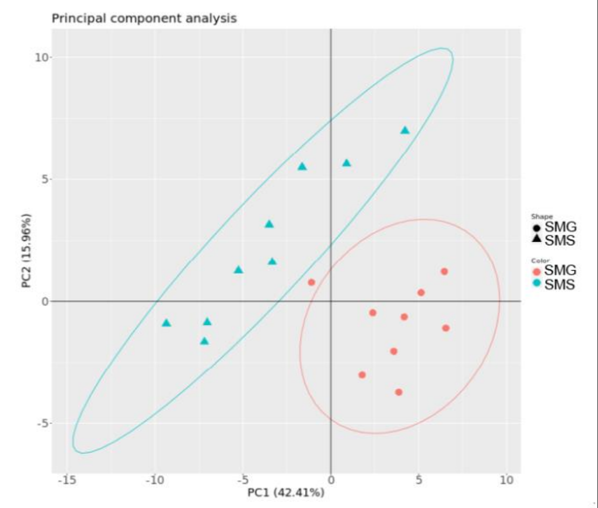

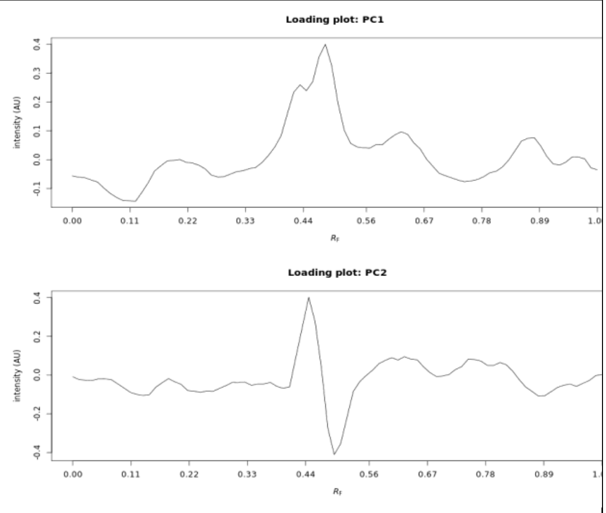

b

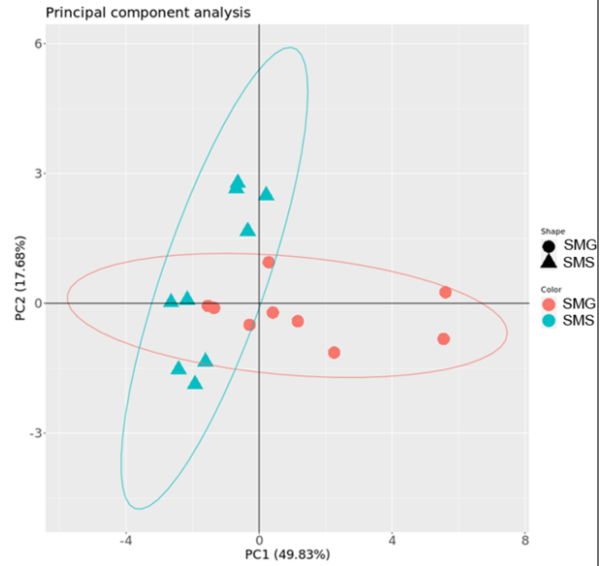

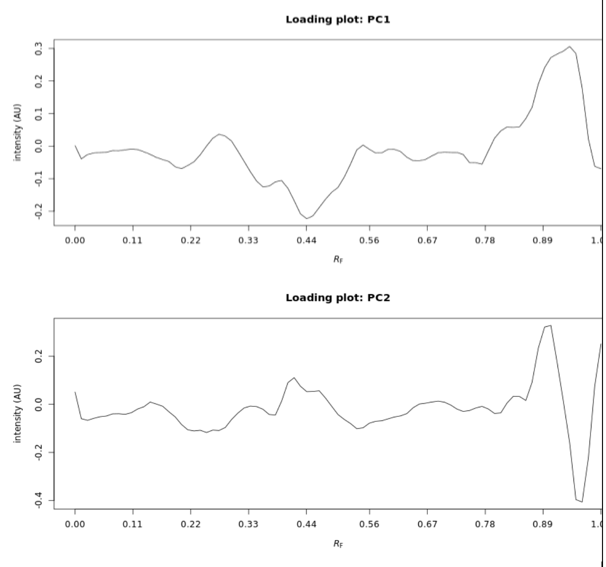

c

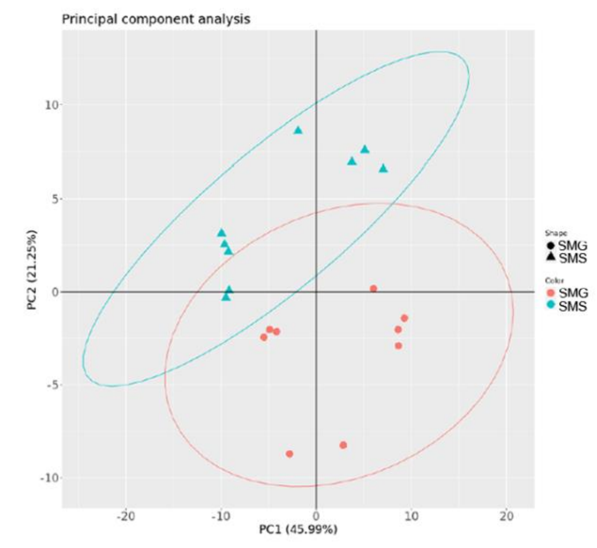

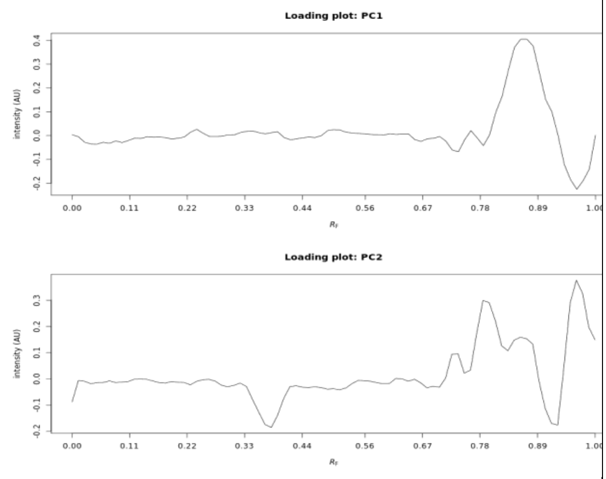

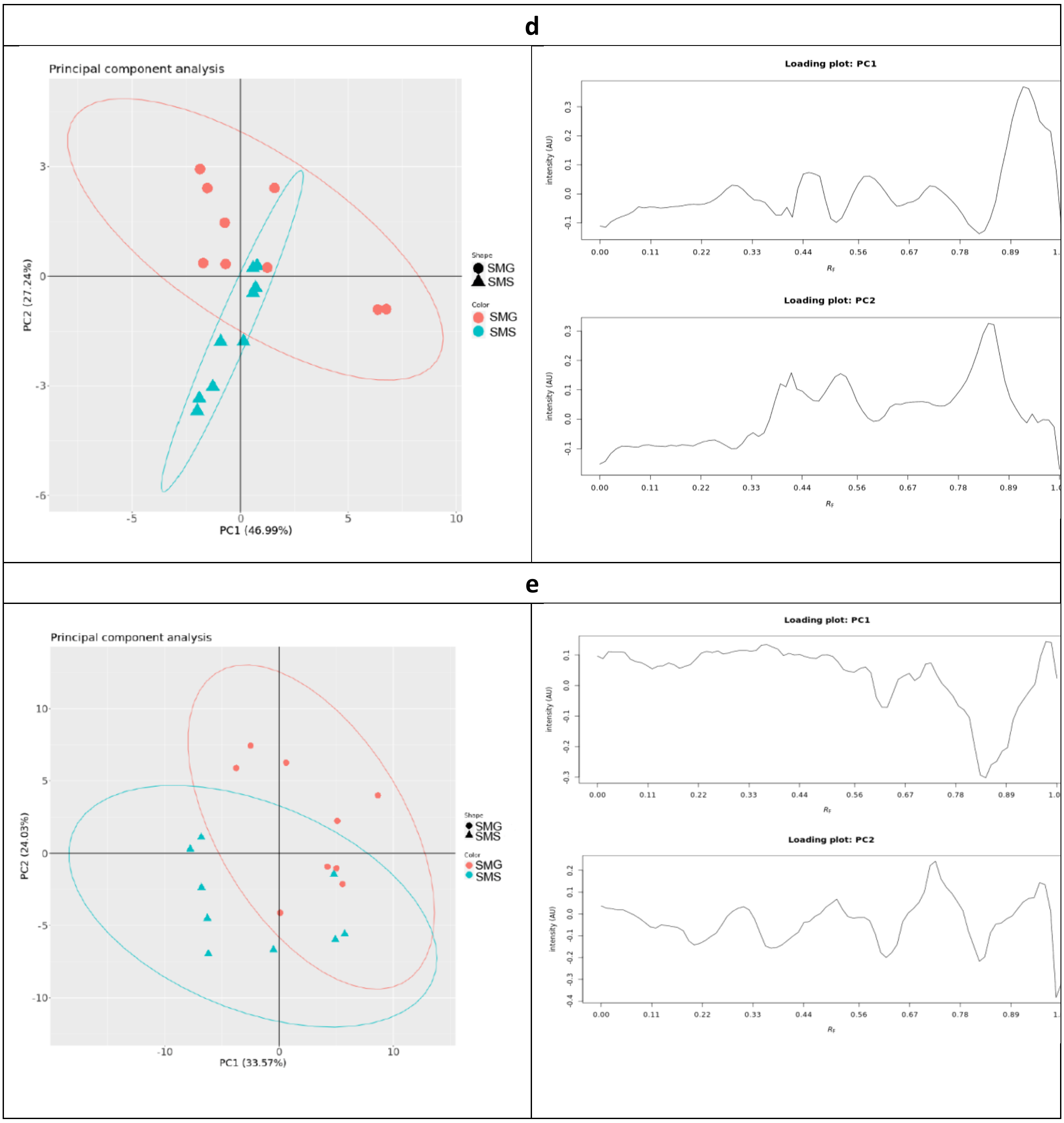

Figure S2. PCA score and loading plots of apple juice extracts performed on profile data obtained from the (**a**) DPPH• assay, (**b**) AChE, (**c**) BChE and (**d**) tyrosinase inhibition assay as well as (e) *Aliivibrio fischeri* bioassay autograms.

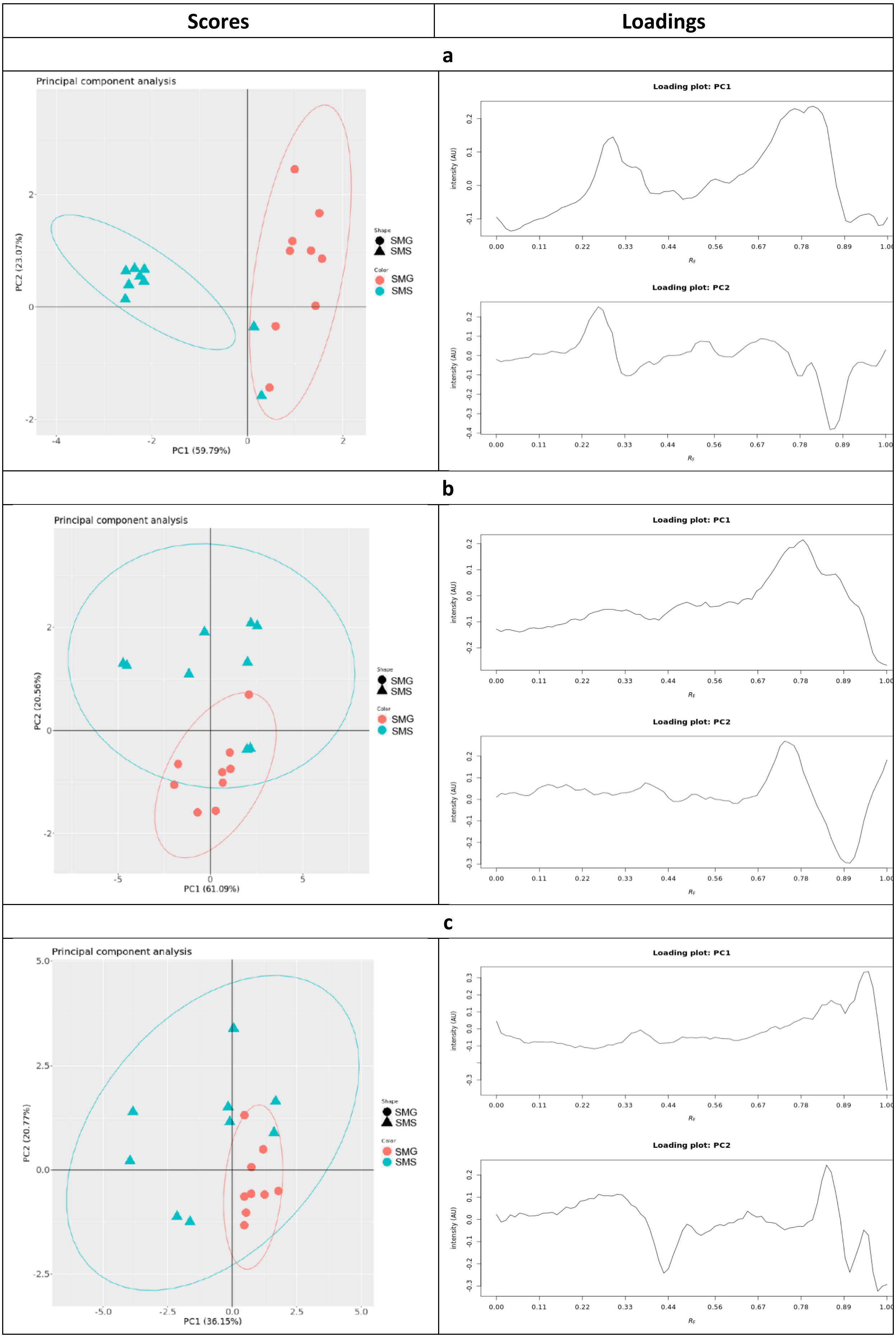
Scores
Loadings
a
Principal component analysis
PC1 (59.79%)
PC2 (23.07%)
Shape
SMG
SMS
Color
SMG
SMS
Loading plot: PC1
Loading plot: PC2
intensity (AU)
0.00 0.11 0.22 0.33 0.44 0.56 0.67 0.78 0.89
b
Principal component analysis
PC1 (61.09%)
PC2 (20.56%)
Shape
SMG
SMS
Color
SMG
SMS
Loading plot: PC1
Loading plot: PC2
intensity (AU)
c
Principal component analysis
PC1 (36.15%)
PC2 (20.77%)
Shape
SMG
SMS
Color
SMG
SMS
Loading plot: PC1
Loading plot: PC2
intensity (AU)

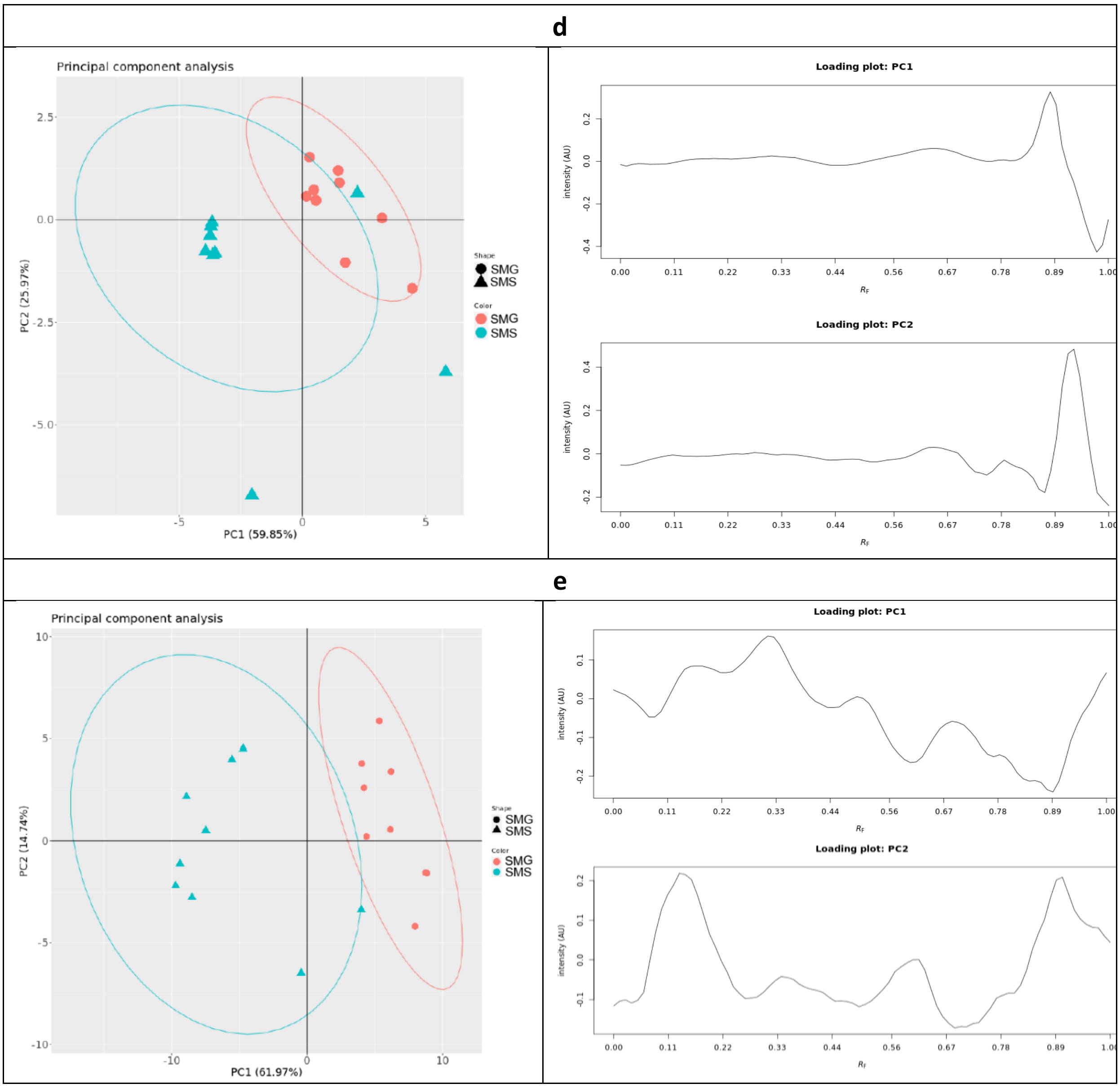

Figure S3. PCA score and loading plots of grape juice extracts performed on profile data obtained from the (**a**) DPPH• assay, (**b**) AChE, (**c**) BChE and (**d**) tyrosinase inhibition assay as well as (**e**) *Aliivibrio fischeri* bioassay autograms.
